# Cancer Diagnostic and Predictive Biomarkers 2018

**DOI:** 10.1155/2019/3879015

**Published:** 2019-01-08

**Authors:** Franco M. Buonaguro, Patrizia Caposio, Maria Lina Tornesello, Valli De Re, Renato Franco

**Affiliations:** ^1^Unit of Molecular Biology and Viral Oncology, Department of Research, Istituto Nazionale Tumori IRCCS Fondazione Pascale, 80131 Napoli, Italy; ^2^Vaccine and Gene Therapy Institute, Oregon Health & Science University, Beaverton, OR 97006, USA; ^3^Immunopathology and Cancer Biomarkers/Bio-Proteomics Facility, Centro di Riferimento Oncologico, IRCCS, 33081 Aviano, Italy; ^4^Division of Pathology, Department of Mental and Physical Health and Preventive Medicine, Università degli Studi della Campania “Luigi Vanvitelli”, 80131 Napoli, Italy

Substantial advances have been achieved in the knowledge of the complex mechanisms regulating development and progression of human cancers. The employment of high throughput techniques allowed the identification of deregulated pathways and altered cellular molecules, including mutation signatures, noncoding RNAs, protein expression profiles, and metabolites, which have a significant impact in the early diagnosis, choice of treatment, and prediction of therapeutic response in many cancer types.

This special issue comprises relevant articles focused on the identification of innovative cancer biomarkers based on the analysis of genes containing driver somatic mutations, the quantitation of microRNAs expression levels, and the characterization of gene expression profiles in different types of cancer. Moreover, several authors investigated the efficacy of circulating biomarkers and molecular imaging techniques for noninvasive diagnosis of cancers.

C. Omarini et al. in Italy in the paper titled “Mutational Profile of Metastatic Breast Cancer Tissue in Patients Treated with Exemestane Plus Everolimus” investigated the mutational status of breast cancer biopsies in patients treated with Exemestane and Everolimus and observed that mutations affecting the PI3K/AKT/mTOR pathway were significantly associated with Everolimus resistance and disease relapse. K. Nie et al. in China in the paper titled “Mutational Profiling of Non-Small-Cell Lung Cancer Resistant to Osimertinib Using Next-Generation Sequencing in Chinese Patients” performed a next-generation sequencing analysis in non-small-cell lung cancer (NSCLC) cases resistant to Osimertinib to identify somatic mutations and new therapeutic targets. The authors observed that EGFR C797S/G and EGFR T790M mutations were the most common in Osimertinib resistant NSCLC patients. A. López-Cortés et al. in Ecuador in the paper titled “Mutational Analysis of Oncogenic AKT1 Gene Associated with Breast Cancer Risk in the High Altitude Ecuadorian Mestizo Population” analyzed the distribution of polymorphic variants of the AKT1 gene in women of the Mestizo population affected by breast cancer and living at high altitudes. The authors found a possible association between the intronic variant rs3803304 GG and the risk of breast cancer in the Mestizo population.

A large number of short noncoding micro RNAs (miRNAs) have been shown to bind the 3'UTR of mRNAs and to selectively deregulate protein translation in many types of cancer. M. Jiang et al. in China in the paper titled “Clinically Correlated MicroRNAs in the Diagnosis of Non-Small Cell Lung Cancer: A Systematic Review and Meta-Analysis” performed a meta-analysis of 71 studies to evaluate the importance of miRNA levels in the diagnosis of NSCLC. They observed that multiple miRNAs have higher diagnostic value than single miRNA in all populations included in the study (Asian, Caucasian, and African populations). The authors concluded that some miRNAs, such as miR-21 and miR-210, could be used as diagnostic biomarkers for NSCLC. J. Pan et al. in China in the paper titled “HSP90: A Novel Target Gene of miRNA-628-3p in A549 Cells” investigated the effect of miR-628-3p on migration and apoptosis of A549 cells. They performed in silico analysis to identify the heat shock protein 90a (HSP90) gene as target of the miR-628-3p in lung cancer. Then they confirmed by molecular studies that miR-628-3p promotes apoptosis and inhibits migration in A549 cells and concluded that miR-628-3p could be a novel strategy for lung cancer treatment.

The study of gene expression by microarray allows simultaneously comparing the transcription of thousands of genes in cancer versus normal tissues. N. Hauptman et al. in Slovenia in the paper titled “Bioinformatics Analysis Reveals Most Prominent Gene Candidates to Distinguish Colorectal Adenoma from Adenocarcinoma” performed an in silico study of gene expression datasets and identified biomarkers able to distinguish the colorectal adenoma, characterized by pseudoinvasion, from early adenocarcinomas. They identified 16 genes differentially expressed (COL12A1, COL1A2, COL3A1, DCN, PLAU, SPARC, SPON2, SPP1, SULF1, FADS1, G0S2, EPHA4, KIAA1324, L1TD1, PCKS1, and C11orf96) which may be used to discriminate colorectal adenoma from carcinoma. Moreover, A. Peng et al. in the paper titled “A Bioinformatic Profile of Gene Expression of Colorectal Carcinoma Derived Organoids” compared the gene expression profiles of colorectal organoids with those of colorectal cancer biopsies obtained by publicly available datasets. They identified common alterations in WNT, MAPK, TGF-*β*, SHH, ECM-receptor interaction, and FGF pathways suggesting that organoids recapitulate original colon cancer tissues.

N. Sekiguchi et al. in Japan in the paper titled “Gene Expression Profile Signature of Aggressive Waldenström Macroglobulinemia with Chromosome 6q Deletion” performed a microarray analysis to investigate the differences in gene expression in the Waldenström macroglobulinemia with and without the chromosome 6q deletion. Their analysis suggested that the BCR signaling pathway and IL21R expression were activated in 6q del cases. Moreover, FOXP1 and CBLB were found to be positive regulators of the BCR pathway with consequences on the aggressiveness of the Waldenström macroglobulinemia with 6q del expression signature. G. Ukmar et al. in Italy in the paper titled “PATRI, a Genomics Data Integration Tool for Biomarker Discovery” developed a bioinformatics tool named “Platform for the Analysis of TRanslational Integrated data” (PATRI) conceived for the integration of clinical and genomics data. The PATRI workflow identifies statistically significant baseline genomics signatures, sensitive and resistant preclinical models, and then prediction of drugs treatment sensitivity in clinical samples.

Several studies have shown that specific biomarkers are released by cancer tissues in the body fluids, such as blood serum, thus reflecting the molecular spectrum of the tumor cells. Y. Kim et al. in Republic of Korea in the paper titled “A Comparative Study for Detection of EGFR Mutations in Plasma Cell-Free DNA in Korean Clinical Diagnostic Laboratories” performed a quality-assurance pilot study to harmonize the testing of circulating tumor DNA among laboratories. The authors observed that the cobas assay was a useful method for detecting EGFR mutations in plasma circulating free DNA and that there is a need for each laboratory to optimize the performance to meet the clinical utility.

H. Zhu et al. in Australia in the paper titled “Cystathionine *β*-Synthase in Physiology and Cancer” reviewed recent literature on the physiological functions of cystathionine *β*-synthase, which plays multifunctional roles in the regulation of cellular energetics, redox status, DNA methylation, and protein modification in cancer cells. They discussed the possible use of cystathionine *β*-synthase and its key metabolites, such as homocysteine, as biomarkers for cancer diagnosis or therapeutic targets. E. Lubowicka et al. in Poland in the paper titled “Plasma Chemokine CCL2 and Its Receptor CCR2 Concentrations as Diagnostic Biomarkers for Breast Cancer Patients” evaluated the plasma levels of CCL2, CCR2, and tumor marker CA 15-3 in breast cancer patients and controls and their findings suggested that CCL2 and CCR2 may be used in the diagnosis of breast cancer in conjunction with CA 15-3. O. Kurtenkov et al. in Estonia in the paper titled “The Thomsen-Friedenreich Antigen-Specific Antibody Signatures in Patients with Breast Cancer” analyzed the serum samples of breast carcinoma patients for the levels of Thomsen-Friedenreich antigen-specific antibody isotypes considering that the serum total immunoglobulins glycosylation has been shown to have a diagnostic potential for some type of cancer. The authors demonstrated that the levels of anti-Thomsen-Friedenreich antigen antibodies, their sialylation profile, isotype distribution, and avidity displayed specific changes that could be used as noninvasive Ab-based biomarkers for early detection of breast cancer. A.-M. Enciu et al. in Romania in the paper titled “*Targeting CD36 as Biomarker for Metastasis Prognostic: How Far from Translation into Clinical Practice?*” in their review summarized many pieces of data to support the role of CD36 as a potential prognostic biomarker in cancer. CD36 is a scavenger receptor for fatty acid uptake which modulates cell-to-extracellular matrix attachment, stromal cell fate (for adipocytes, endothelial cells), TGF*β* activation, and immune signaling. It has been proposed as a prognostic marker in various cancers of epithelial origin. A. Arasu et al. in India in the paper titled “PAX3: A Molecule with Oncogenic or Tumor Suppressor Function Is Involved in Cancer” reviewed current literature on the activity of the transcription factor PAX3/Pax3, which contributes to diverse cell lineages during embryonic development and plays a major role in tumorigenesis. The authors highlighted the oncogenic and tumor suppressor role of PAX3 in different cancer types. C. A. Salter et al. in USA in the paper titled “Alkaline Phosphatase Kinetics Predict Metastasis among Prostate Cancer Patients Who Experience Relapse following Radical Prostatectomy” studied the alkaline phosphatase velocity in order to predict distant metastasis-free survival in a retrospective cohort of prostate cancer patients. They observed that rapid alkaline phosphatase velocity was a strong predictor of distant metastasis-free survival.

Predicting the cancer outcome from histological analysis may provide guidance for surgeons and oncologists on the appropriate treatment, particularly in developing countries. B. S. M. S. Siriwardena et al. in Sri Lanka in the paper titled “A Predictive Model to Determine the Pattern of Nodal Metastasis in Oral Squamous Cell Carcinoma” have developed a histological prediction model to estimate the probability of developing metastasis in patients affected by oral cancer. They performed a multivariate analysis and observed that the level of differentiation, the pattern of invasion, and stage are the most important predictors of metastasis in oral cancer. S. H. Kim et al. in Republic of Korea in the paper titled “Effect of Neoadjuvant Hormone Therapy on Resection Margin and Survival Prognoses in Locally Advanced Prostate Cancer after Prostatectomy Using Propensity-Score Matching” investigated the effect of neoadjuvant hormone therapy on resection margin positivity, biochemical-recurrence-free survival, and overall survival in patients with locally advanced prostate cancer treated with radical prostatectomy using propensity-score matching. They concluded that neoadjuvant hormone therapy was not a significant factor for resection margin positivity in these patients.

Molecular imaging enables the in vivo visualization and quantification of biologic processes in normal tissues and cancers. M.-C. Shih et al. in Brazil in the paper titled “Efficient Synthesis of Glutamate Peptide-Estradiol Conjugate for Imaging Estrogen Receptor-Positive Diseases” used radiolabelled estrogen receptor ligand to quantify estrogen receptor tissue uptake for staging and restaging of cancers as well as endometriosis. The in vivo PET imaging studies indicated that 68Ga-GAP-EDL could identify estrogen receptor positive tumors in MCF-7 tumor-bearing mice. A. P. Burlaka et al. in the paper titled “Rectal Cancer: Redox State of Venous Blood and Tissues of Blood Vessels from Electron Paramagnetic Resonance and Its Correlation with the Five-Year Survival” analyzed the redox state of venous blood and tissues in patients with rectal cancer by the spin-trapping electron paramagnetic resonance measuring the intensity of the signals from ceruloplasmin, transferrin, and labile iron pool. The results showed that the intensities of the signals from the “native” and “trapped” paramagnetic centres can be potentially used for the study of the rectal cancer molecular mechanisms. D. M. Yeo et al. in Republic of Korea in the paper titled “Histogram Analysis of Perfusion Parameters from Dynamic Contrast-Enhanced MR Imaging with Tumor Characteristics and Therapeutic Response in Locally Advanced Rectal Cancer” performed histogram analysis of perfusion parameters from dynamic contrast-enhanced magnetic resonance imaging (DCE-MRI) evaluating the entire tumor volume and correlated with EGFR expression, KRAS mutation, and therapy response to determine the overall tumor characteristics in rectal cancer. A. Bevilacqua et al. in Italy in the paper titled “CT Perfusion in Patients with Lung Cancer: Squamous Cell Carcinoma and Adenocarcinoma Show a Different Blood Flow” characterized the tumor baseline blood flow in two lung cancer subtypes, the adenocarcinoma and squamous cell carcinoma. They observed a different hemodynamic behaviour between adenocarcinoma and squamous cell carcinoma, suggesting that such parameter could be considered as a biomarker supporting treatment planning to select the patients that would benefit from antiangiogenic therapies.

